# Occupational exposure to ultrafine particles in police officers: no evidence for adverse respiratory effects

**DOI:** 10.1186/s12995-018-0187-8

**Published:** 2018-02-03

**Authors:** G. Jordakieva, I. Grabovac, E. Valic, K. E. Schmidt, A. Graff, A. Schuster, K. Hoffmann-Sommergruber, C. Oberhuber, O. Scheiner, A. Goll, J. Godnic-Cvar

**Affiliations:** 1 University Department of Physical Medicine, Rehabilitation and Occupational Medicine, Medical University of Vienna, Waehringer Guertel 18-20, A-1090 Vienna, Austria; 2 Department of Social and Preventive Medicine, Centre for Public Health, Medical University of Vienna, Kinderspitalgasse 15/1, A-1090 Vienna, Austria; 3 Austrian Worker’s Compensation Board (AUVA), HUB, Adalbert-Stifter-Straße 6, 1200 Vienna, Austria; 4 University Clinic of Internal Medicine II, Institute of Occupational Medicine, Medical University of Vienna, Waehringer Guertel 18-20, A-1090 Vienna, Austria; 5 Austrian Dust-Silicosis Control Center (ÖSBS), Einödmayergasse 12, 8700 Leoben, Austria; 6 Department of Pathophysiology and Allergy Research, Medical University of Vienna, Waehringer Guertel 18-20, A-1090 Vienna, Austria; 7 Section of Medical Statistics, Core Unit for Medical Statistics and Informatics, Medical University of Vienna, Waehringer Guertel 18-20, A-1090 Vienna, Austria

**Keywords:** Adverse health effects, Airway obstruction, Inflammation, Occupational exposure, Ultrafine particles, Indoor shooting range

## Abstract

**Background:**

Inhalation exposure to fine and ultrafine particles (UFPs) has been associated with respiratory diseases. However, little is known on the quality, threshold levels and concentration of these particles causing adverse health effects.

**Methods:**

The impact of occupational exposure to submicrometer and UFPs was assessed in 30 healthy police shooting instructors by clinical investigation, self-assessment questionnaire, sputum and spirometry and compared to a control group. General laboratory chemistry parameters, circulating cytokines (interleukin [IL]-2, IL-4, IL-5, IL-6, IL-8, interferon-gamma [IFN-γ]), and granulocyte macrophage colony-stimulating factor (GM-CSF) in serum were measured. UFP exposure was recorded by Scanning Mobility Particle Sizer.

**Results:**

Concentrations of submicrometer sized airborne particles (< 700 nm) measured between 3.34 × 10^5^/cm^3^ and 7.58 × 10^5^/cm^3^ at shooting sites, with highest concentrations found in the UFP range (< 100 nm). The size of the monodispersed particles ranged from 54.74 ± 16.25 nm to 98.19 ± 22.83 nm. Short term exposure (4 h) to high levels of UFPs caused an increase of IFN-γ in exposed subjects (*p* = 0.022). 24 h after exposure a significant decrease of IgG, albumin fibrinogen and factor VII was found. Neither directly after 4 h of high levels UFPs exposure nor 24 h after exposure subjective complaints or objective measurements indicating adverse respiratory effects in exposed subjects were found.

**Conclusions:**

No consistent indications for adverse respiratory or inflammatory effects directly following exposure and 24 h after exposure to high levels of UFPs in our study group were detected. However we showed the assessment of short-term exposure effects at a genuine occupational setting, which might is relevant when a risk assessment of high level occupational exposures to UFPs is considered.

## Background

A relationship between increased ambient air pollution and adverse health effects has been postulated. Exposure to ultrafine particles (UFPs, defined as < 100 nm [nm] in diameter) has been reported to increase respiratory symptoms, decrease lung function, cause asthma exacerbations, increased medication use and increased hospital admissions related to respiratory diseases [1–7]. UFPs predominantly emerge from industrial (welding and metal smoke, technical carbon-particulate emissions, amorphous silica acid) and traffic emissions. Their adverse health effects have been attributed to high particle number per unit mass and reactive surface [7–13] and associated with airway inflammation with a subsequent release of chemical mediators and cytokines [14–18]. Adjuvant effects have also been observed, e.g. diesel particles have been shown to enhance and/or intensify sensitization to common allergens [9, 11, 12, 19–26].

Most of the current knowledge on pathophysiological mechanisms associated with UFPs is derived from cell cultures and animal models. Only a few groups investigated their effects on human subjects [7–10, 12–14, 16–21, 23–25, 27, 28].

While acute respiratory effects of exposure to fine particles have been established experimentally and epidemiologically, the contribution of fine particles to the development of chronic disease is less clear [29].

The measurement of UFPs at workplaces in Germany has shown that especially high number concentrations (ranging from a few 1000 to several 100,000 particles/cm^3^) are associated with combustion processes such as; welding, soldering and metal grinding [30]. Firearm usage generates particulate emissions, which may accumulate in ambient air, particularly in closed spaces such as indoor shooting ranges. Airborne particles originate from explosion and vapour generation during bullet discharge, further releasing metallic components, like lead, barium and antimony. Particle diameters emitted from gunshots were reported to be mostly < 10 μm in diameter [31]. Work-site measurements conducted at indoor shooting ranges revealed a clear accumulation of UFPs ranging up to 420,000/cm^3^ in Swiss and German workplace assessments, depending on exhaust availability [32, 33].

It remains to be established whether exposure to submicrometer and UFPs triggers adverse respiratory effects only in susceptible or in all exposed subjects. Even more so, adverse health effects of UFPs in an occupational setting after several years of exposure have not been evaluated thoroughly.

The aim of this study was to determine whether a defined occupational exposure to UFPs causes adverse health effects in frequently inhalation exposed shooting instructors.

## Methods

### Subjects

Sixty male subjects (*n* = 60) employed as federal police officers in Vienna, Austria, were included in this study and divided in two groups depending on the level of UFP exposure. The exposed group consisted of 30 police officers working as instructors at a shooting range with an average work history of 4.7 years. The control group comprised 30 police officers working in administration.

### Study design

Measurements in subjects were conducted at a) three time points in the exposed group: time point “0^exp^” = before exposure, time point “1^exp^” = at the day of exposure (after 4 h of UFP exposure) and time point “2^exp^” = 24 h after UFP exposure; and at b) two time points in the control group (no occupational UFP exposure) on two separate days (time point “0^con^” and time point “1^con^”). At each of these test points an occupational self-reporting questionnaire was completed and a diagnostic workup performed (spirometry and blood sampling).

### Exposure to UFPs and metals

Shooting ranges have been identified as places with high levels of submicrometer and UFPs [32, 34, 35]. The measurements were conducted at 5 modern police shooting ranges (designated as “A”, “B”, “C”, “D” and “E”), where UFPs arise from combustion of the explosion in the percussion-cap and metal fumes originating from the metal coat of the bullets (lead, barium, copper and antimony).

The average size of airborne particles (in nm), the metal content (in mg/m^3^) of the inhalable dust and the gaseous pollutants were measured at 5 locations (Table 2). At location A and B the measurements were repeated twice and three times, respectively, on separate days to control for the repeatability of the measured values. The number and size of submicrometer and UFPs under normal working conditions while the ventilation (exhaust) system was operating (air turnover between 22 and 31 air changes/h, air flow from ceiling to target plates) was measured by the stationary Scanning Mobility Particle Sizer (SMPS™, TSI Type 3936 L; Flow Controller Model 120FC TSI; impactor diameter 0,0457 cm) and expressed as particles/cm^3^ (concentrations indicated as means +/− SD). Measurement range was restricted to particle sizes 15–700 nm and measured values were presented every 2 min and 30 s.

Sampling was conducted between 2 and 4 h (see Table 2) in a total of eight shooting episodes, during which constant target practice was performed by up to 3 persons. The distance between SMPS and shooting person as well as between sampling inlet and pistol outlet were less than a meter, respectively. The study subjects, being shooting instructors, were constantly present at the shooting range and in close proximity to the shooting persons.

All aerosol exposure measurements were performed under normal working conditions with an activated ventilation system. The different particle sizes were determined using a high-voltage area resulting from particle diameter-dependent mobility of the submicrometer and ultrafine aerosol particles in the surrounding gas. First of all the aerosol passes an impactor, which prevents larger particles than those of interest arriving in the system. Subsequently, ionization of the particles takes place using a Kr^87^ source. In a high-voltage area (DMA 3081 Differential Mobility Analyser) the particles are selected according to their electrical mobility, which is a measure for their size. Mono-dispersive aerosol is supplied to a CPC (Condensation Particle Counter 3022A), where the particles are brought to the same size with the help of evaporating butanol, in order to be able to count them using a laser. The entirely controlling of the SMPS as well as the data measurement recording was conducted by PC.

Inhalable dust was measured by the personal air sampler (Gilian HFS 513 A Gilian - PAS SG10, GSA building of measure devices GmbH using a standard kit; Filter type: 10 μ nylon; holder: “O-ring seal”), the high volume sampler Gravikon PM4 (Ströhlein) and the portable dust monitor (Grimm Aerosol Technik GmbH using standard equipment). Personal sampling time was approximately 3 h. Measurements were performed 6 m and 8 m away from the shooter and 0 and 2 m away from the targets; additionally personal sampling was conducted on the shooting instructors themselves. The variability of these measurements was below 6% (calculated by GRIMM Aerosol Technik GmbH Windows).

Following gaseous pollutants: CO, NO, NO_2_ - were measured by the gas monitor VRAE (RAE Systems). At ranges “A” and “B” the measurements were repeated twice and three times respectively.

To estimate baseline (non-occupational) exposure to UFPs we measured particles with the same methods at a nearby school twice for 16 h (mainly at nighttime).

### Spirometry

Lung function was assessed by FVC, FEV_1_, MEF_50_ and MEF_25_ measurements (American Thoracic Society 1995) with a “Flow Screen Pro” spirometer (Jäger, Germany) according to the ATS criteria [36].

### Blood sample analysis

Blood was drawn from periphery veins of participating subjects. After centrifugation (4000 rpm/10 min/room temperature) serum samples were stored at − 20 °C. Two kinds of analyses were performed:

- Blood cell count (total and differential), biochemical parameters (albumin, C-reactive protein [CRP]), haemostasis parameters (fibrinogen, prothrombin test, coagulation factor VII), immunoglobulins (IgA, IgG, IgE), and lead concentrations in blood were measured in a clinical laboratory.
- Cytokines (interleukin (IL-)2, IL-4, IL-6, IL-8, interferon (IFN-)γ, granulocyte macrophage colony-stimulating factor (GM-CSF)) were assessed by commercial ELISA (enzyme-linked immunosorbent assay) kits (R&D Systems, Inc., Minneapolis, USA) according to manufacturer’s instructions following the construction of standard curves for each ELISA system. All serum samples were applied in duplicates and undiluted. The tests were performed in an immunological laboratory of the Department of Pathophysiology and Allergy Research of the Medical University of Vienna.

### Statistical methods

This study was planned as a feasibility study. Descriptive statistics (percentages, means, ranges and standard deviation) were calculated. To investigate the differences in values between the two groups at baseline (before exposure) t-tests, Wilcoxon tests or Chi Square tests were performed as appropriate.

To analyse a possible temporal impact of exposure to submicrometer and UFPs, analyses of covariance was performed for the first measurement after baseline (which is available in exposed and controls) including a fixed grouping factor (exposed/controls) and the baseline value, age and package years as co-variables. A significant grouping factor here indicated a different time course between exposed subjects and controls.

Only within the group of exposed persons, mixed models accounting for time as fixed factor (with three levels) and exposed subjects as a random factor were calculated. No correction for multiple testing was applied in this pilot study. Statistical analyses were performed using SAS Version 9.1.

## Results

### Demographic data

A total of sixty (*n* = 60; 100% male subjects) police officers with (*n* = 30) and without (*n* = 30) occupational UFP exposure were examined. The exposed group consisted of 30 police officers working as instructors at a shooting range with an average work history of 4.7 years. The control group comprised 30 police officers working in administration. Control group subjects were older (41.9 vs. 34.1 years, respectively; *p* < 0.0001) and had a longer (13.17 vs. 6.83 years, respectively; *p* = 0.020) and more intensive (14.47 vs. 5.53 package years, respectively; *p* = 0.014) smoking history than the exposed subjects (Table 1).

**Table 1:** Characteristics of 30 shooting instructors occupationally exposed to UFPs for 4.7 years on average and 30 control participants

	Exposed to UFPs	Controls	*p*-value (t-test)
Age, years (mean)	34.1 ± 8.0	41.9 ± 5.1	< 0.0001
Current smokers %	29	34	0.65^b^
Ex-smokers %	29	31	0.87^b^
Years	6.83 range (0–25)	13.17 range (0–36)	0.02^a^
Pack-years	5.53 range (0–30)	14.47 range (0–60)	0.01^a^
Body height in cm	179.3 ± 5.5	181.6 ± 7.3	0.18
Body weight in kg	80.5 ± 11.2	84.5 ± 13.2	0.08
Systolic BP in mmHg	128.0 ± 14.1	128.0 ± 14.9	0.83
Diastolic BP in mmHg	82.3 ± 10.6	82.8 ± 10.1	0.71
VC (L)	5.01 ± 0.93	5.45 ± 0.97	0.0165
FVC %	95.6 ± 15.6	90.1 ± 13.4	
FEV_1_%	96.8 ± 13.2	92.8 ± 17.0	
MEF_50_ (%)	94.7 ± 25.7	90.0 ± 32.0	
MEF_25_ (%)	85.7 ± 34.8	72.3 ± 36.8	

Data expressed in mean ± standard deviation

*BP* blood pressure, *VC* vital capacity, *FEV*_*1*_ forced expiratory volume in one second, *MEF*_*50*_ maximum expiratory flow at 50% of forced VC, *MEF*_*25*_ maximum expiratory flow at 25% of forced VC

^a^Wilcoxon test

^b^ChiSquare test

### UFPs exposure load

The size of monodispersed particles ranged from 54,74 ± 16,25 nm (SD) nm at location A1 to 98.19 ± 22.83 (SD) nm at location E (Table 2). The metal content of inhalable dust was on average as follows: lead 0.01 ± 0.2 (SD), barium 0.05 ± 0.04 (SD), copper 0.01 ± 0.01 (SD), and antimony 0.002 ± 0.01 (SD). Exposure to gaseous pollutants was neglectable: CO 4.86 ± 4.87 (SD) ppm, NO and NO_2_ expressed as NOx was < 0.1 ppm. Baseline (non-occupational) measurements at the control site with the same methods (nearby school) were 0.007 and 0.009 mg/m^3^ for inhalable dust, whereas the metal content on average was as follows: lead 0.02 μg/m^3^, barium 0.02 μg/m^3^ and copper 0.5 μg/m^3^.

**Table 2:** Exposure to submicrometer sized airborne particles < 700 nm during 4,5 h of the work shift at 5 different locations

Location	Duration (in hours)	AM particle concentration (n/cm^3^)	Median particle size (nm)	Mean particle size (nm)	Dominating Mode (nm)
A1	3.5	3.57 × 10^5^	54.7	67.2	56.6
A2	3.5	5.31 × 10^5^	62.3	72.8	63.3
B1	3.5	4.66 × 10^5^	58.8	70.1	60.1
B2	3.5	5.93 × 10^5^	60.3	70.3	64.5
B3	4	5.39 × 10^5^	62.1	72.3	65.8
C	3.5	4.28 × 10^5^	62.8	75.4	63.4
D	3.5	7.58 × 10^5^	78.1	89.2	83.3
E	2	3.34 × 10^5^	98.2	109.8	101.0

A-E: different locations, at location A and B the measurements were repeated on 2 and 3 study days, respectively, in parallel to the medical examinations

*AM* arithmetic mean

### Self-reported symptoms

No health complaints, neither local (airways) nor systemic, were reported acutely (after 4 h of UFP exposure) or belatedly (24 h after exposure). Subjects who were occupationally exposed to submicrometer and UFPs did not report more airway or general health complaints at baseline than subjects in the control group.

### Spirometry

- At baseline

Baseline spirometry values significantly differed between the exposed and non-exposed subjects. Baseline vital capacity (VC) values in control subjects were lower than the baseline VC values in exposed subjects (5.01 ± 0.93 [SD] L vs. 5.45 ± 0.97 [SD] L, respectively, *p* = 0.0165) as shown in Table 1. In the exposed shooting instructors a significant reduction of the FEV_1_ lung function parameter (for 0.23 L on average *p* = 0.0104) was found only in the 24 h after exposure spirometry when compared to baseline values. No reduction in MEF_50_ or in MEF_25_ was found at any time point (Tables 3 and 4) in exposed subjects. In the control group no significant difference between time point 0^con^ and time point 1^con^ in FEV_1_ (*p* = 0.5517), MEF_50_ (*p* = 0.7302) and MEF_25_ (*p* = 0.6537) was found.

- After 4 h of UFP exposure and 24 h after exposure

**Table 3:** Lung function in shooting instructors occupationally exposed to UFPs (3 study points)

	Timepoint 0	Timepoint 1	Timepoint 2
VC (L)	5.5 ± 1.0	5.3 ± 0.9	5.4 ± 0.7
VC (%)	95.6 ± 15.5	93.2 ± 16.2	94.6 ± 13.5
FEV_1_ (L)	4.6 ± 0.7	4.5 ± 0.7	4.3 ± 0.6
FEV_1_ (%)	96.8 ± 13.2	95.0 ± 11.2	92.5 ± 10.5
MEF_50_ (L/s)	2.8 ± 0.8	2.8 ± 0.8	2.6 ± 0.7
MEF_50_ (%)	94.7 ± 25.7	95.8 ± 25. 7	89.6 ± 23.0
MEF_25_ (L/s)	1.9 ± 0.3	2.0 ± 0.2	2.0 ± 0.2
MEF_25_ (%)	85.7 ± 34.8	82.2 ± 32.9	77.2 ± 27.2

Data expressed in mean ± standard deviation

VC, vital capacity; FEV_1_, forced expiratory volume in one second; MEF_50_, maximum expiratory flow at 50% of forced VC; MEF_25_, maximum expiratory flow at 25% of forced VC

**Table 4:** Lung function of control participants (2 study points)

	Timepoint 0	Timepoint 1
VC (L)	5.0 ± 0.9	5.1 ± 0.8
VC (%)	90.1 ± 13.4	92.6 ± 12.8
FEV_1_ (L)	4.2 ± 0.8	4.1 ± 0.5
FEV_1_ (%)	92.8 ± 17.0	91.15 ± 10.1
MEF_50_ (L/s)	2.4 ± 0.7	2.4 ± 0.7
MEF_50_ (%)	90.0 ± 11.9	87.1 ± 29.2
MEF_25_ (L/s)	1.7 ± 0.3	1.7 ± 0.3
MEF_25_ (%)	72.3 ± 36.8	69.2 ± 26.8

Data expressed in mean ± standard deviation

*VC* vital capacity, *FEV*_*1*_ forced expiratory volume in one second, *MEF*_*50*_ maximum expiratory flow at 50% of forced VC, *MEF*_*25*_ maximum expiratory flow at 25% of forced VC

No significant differences in lung function parameters were found after 4 h of UFP exposure between exposed and control subjects when adjusted for baseline value, age and package years. Since measurements after 24 h have only been available for the exposed group we performed a pairwise comparison within the group of exposed subjects. No significant changes were found in lung function parameters, except for the mentioned FEV_1_ decrease 24 h after exposure when compared to baseline FEV_1_.

### Blood cell count and serum parameters

- At baseline

Exposed subjects had higher albumin values at baseline (*p* = 0.004). Apart from that no significant difference between exposed and control subjects was found in complete and differential blood cell count, biochemical parameters, haemostasis factors, immunoglobulins (IgA, IgG, IgE) and in acute phase proteins (C-reactive protein [CRP] and fibrinogen).

- After 4 h of UFP exposure and 24 h after exposure

No difference in measured serum parameters was found after 4 h of UFP exposure in the exposure group and at the second time point in the control group (see Tables 5 and 6) compared to baseline values. 24 h after UFP exposure a slight decrease within exposed subjects was observed for albumin (mean difference: 0.96, *p* = 0.0093), IgG (mean difference 45.3, *p* = 0.0001), coagulation factor VII (mean difference: 4.87; p = 0.009) and fibrinogen (mean difference: 0.8; *p* = 0.003) compared to baseline.

**Table 5:** Biochemical serum parameters in shooting instructors occupationally exposed to UFPs (3 study points)

	Timepoint 0	Timepoint 1	Timepoint 2	*p* value
HDL	51.0 ± 11.6	49.2 ± 11.1	50.6 ± 11.2	n.s.
LDL	121.3 ± 39.8	111.0 ± 33.9	114.0 ± 41.4	n.s.
Cholesterol	201.3 ± 46.3	189.4 ± 44.0	196.2 ± 44.3	n.s.
Albumin	46.8 ± 2.4	46.5 ± 3.2	45.8 ± 2.7	0.0093
IgG	1066.7 ± 180.5	1042.0 ± 183.1	1021.4 ± 170.7	0.0001
FVII	101.9 ± 31.0	99.0 ± 41.7	97.0 ± 33.1	0.009
Fibrinogen	2.5 ± 0.5	2.1 ± 0.9	2.0 ± 0.7	0.003

Data expressed in mean ± standard deviation

*HDL* high-density lipoprotein, *LDL* low-density lipoprotein, *IgG* immunoglobulin G, *FVII* factor VII, *n.s.* non significant

**Table 6:** Biochemical serum parameters of control participants at 2 study points

	Timepoint 0	Timepoint 1	*p* value
HDL	50.2 ± 8.9	53.0 ± 9.5	n.s.
LDL	137.3 ± 35.0	139.7 ± 32.5	n.s.
Cholesterol	220.0 ± 38.3	225.5 ± 38.0	n.s.
Albumin	45.1 ± 1.9	44.8 ± 1.8	n.s.
IgG	1085.1 ± 203.0	1099.5 ± 208.4	n.s.
FVII	110.5 ± 20.2	115.0 ± 18.8	n.s.
Fibrinogen	2.6 ± 0.4	2.7 ± 0.4	n.s.

Data expressed in mean ± standard deviation

*HDL* high-density lipoprotein, *LDL* low-density lipoprotein, *IgG* immunoglobulin G, *FVII* factor VII, *n.s.* non significant

### Cytokine levels in serum

- At baseline

There was no significant difference in cytokine levels in serum (IL-2, IL-4, IL-5, IL-6, IL-8, IFN-γ and GM-CSF) between exposed subjects and controls at baseline (see Tables 7 and 8).

- After 4 h of UFP exposure and 24 h after exposure

**Table 7:** Cytokines in sera from shooting instructors occupationally exposed to UFPs (*N* = 30)

Cytokines	Timepoint 0	Timepoint 1	Timepoint 2
IL-2 ng/ml	4.4 (0–29.9)	4.0 (0–21.8)	5.8 (0–51.8)
IL-4 ng/ml	0.2 (0–3.2)	0.5 (0–2.7)	0.1 (0–1.0)
IL-5 ng/ml	2.2 (0–28.6)	2.7 (0–26.5)	1.4 (0–39.4)
IL-6 ng/ml	0.4 (0–3.1)	0.9 (0–4.3)	0.3 (0–1.7)
IL-8 ng/ml	16.0 (0–73.8)	28.8 (0–244.2)	25.7 (0–172.6)
IFN-γ	9.6 (0–87.3)	25.1 (0–153.2)	10.7 (0–75.1)
GM-CSF	0.6 (0–4.1)	1.0 (0–6.3)	0.8 (0–8.4)

Data expressed in mean (range)

*IL* interleukin, *IFN-γ* interferon gamma, *GM-CSF* granulocyte macrophage colony-stimulating factor

**Table 8:** Cytokines in sera of control participants (*N* = 30)

Cytokines	Timepoint 0	Timepoint 1
IL-2 ng/ml	3.0 (0–33.4)	2.6 (0–34.5)
IL-4 ng/ml	0.02 (0–0.7)	0.5 (0–13.8)
IL-5 ng/ml	7.5 (0–101.9)	4.5 (0–43.4)
IL-6 ng/ml	0.3 (0–3.0)	1.3 (0–26.3)
IL-8 ng/ml	9.6 (0–24.0)	14.0 (0–87.0)
IFN-γ	11.5 (0–89.1)	10.6 (0–53.6)
GM-CSF	0.3 (0–1.9)	0.2 (0–3.4)

Data expressed in mean (range)

*IL* interleukin, *IFN-γ* interferon gamma, *GM-CSF* granulocyte macrophage colony-stimulating factor

No significant changes were found after 4 h of exposure to UFPs except for a significant increase in IFN-γ serum concentration (1.5-fold increase in exposed subjects) (*p* = 0.022, Tables 7 and 8). No significant changes in cytokine levels were found 24 h after UFP exposure.

### Blood metal concentration

A higher blood lead concentration (*p* = 0.0008) was found in shooting instructors than in controls (109.33 ± 103.63 (SD) μg/L vs. 36.24 ± 20.42 μg/L, respectively) at baseline. No significant change in blood lead and other metal concentrations was found following UFP exposure and 24 h after exposure compared to baseline in the exposed group and at both time points in the control group.

## Discussion

Contrary to our expectations, occupational exposure to high doses of submicrometer (< 700 nm) particles, with predominant concentrations in the UFP range, did not cause complaints or significant respiratory effects, neither acutely (after 4 h hours of exposure) nor belatedly (24 h after exposure) in our study subjects working as instructors at shooting ranges.

These findings are remarkable because the amount of UFPs at the presented occupational setting (shooting ranges) reached approximately twice the level of episodic peak environmental concentration of UFPs (up to 300,000/cm^3^ with approximately 50 μg/m^3^ of mass) and belongs to the highest known occupational exposures. UFPs are known to be generally much more biologically aggressive than larger environmental particles, due to their physical properties and their ability to readily cross the alveolo-vascular barrier [23, 37, 38]. The results of our “real life exposure” setting are in concordance with results from controlled laboratory exposures to 500 μg/m^3^ of fine (1.9 × 10^5^ FP/cm^3^, median diameter 291.2 ± 20.2 nm) and UF zinc oxide particles (4.6 × 10^6^ UFPs/cm^3^, median diameter 40.4 ± 2.7 nm) [39].

A possible explanation for the lack of significant pathological observations after exposure to UFPs could be the characteristics of our examined study population, which were predominantly healthy, young and well trained men. Like in other potentially health-adverse occupational environments, the possibility of a “healthy worker effect” could be considered. According to information obtained from the study subjects themselves, however, ex-collaborators who left this particular working environment rarely did so for health related reasons. In currently available scientific literature, the majority of previously documented harmful effects of UFPs in human subjects were shown in susceptible subjects or in patient groups. Those epidemiologic studies have shown that increased particulate air pollution (PM10 and PM2.5) is significantly associated with increased respiratory and cardiovascular morbidity, worsening of asthma, intensified medication, higher hospital admissions and mortality [1–3, 7]. Furthermore another study found associations even between ambient UFPs and mortality. Our study group mainly consisted of healthy and physically fit young men, as mentioned above [4].

Although we found a statistically significant late onset impairment (24 h after exposure) of FEV_1_ air flow, we doubt that it is caused by the exposure to UFPs. Firstly, small airways, which are the target for deposition of fine and ultrafine particles, would be the first to show obstruction in air flow. A matching impairment of MEF_50_ and MEF_25_ which reflect the function of the small airways was not found in our subjects. Secondly, FEV_1_ is, equally to other lung function parameter, influenced by subjects’ motivation and effort; thus a decline in readiness for collaboration cannot be excluded at the final measurement. The interpretation of our results is in accordance with several studies [36, 40, 41] who all failed to show a reduction in lung function parameters after exposure to UFPs. Even in asthmatics and patients with chronic pulmonary disease (COPD) no decrease in lung function after exposure to UFPs could be demonstrated [42–44].

Although UFPs readily cross the alveolo-capillary barrier the chosen circulating inflammatory markers (serum cytokine levels and haemostatic factors, as well as biochemical parameters), did not indicate any systemic reaction neither after 4 h of exposure nor 24 h after occupational UFP exposure [23, 45, 46], except for an increase in circulating IFN-γ levels 4 h after UFP exposure compared to baseline values. IFN-γ is involved in first line innate immune responses to potential pathogens by activation of macrophages [47] and can be released from several cell types, e.g. T cells, natural killer cells and epithelial cells. Immediate increase in IFN-γ levels after UFP exposure has not been previously reported in vivo, however, Huang et al. found that ultrafine carbon particle inhalation in healthy individuals induced inflammation related pathways, e.g. T cell receptor and natural killer cell signalling, which might be an associated mechanism for preformed IFN-γ release [48]. Notably, a significant decrease of serum albumin, immunoglobulin G (IgG), coagulation factor VII, and fibrinogen was measured 24 h after UFP exposure. Albumin, IgG and fibrinogen have been shown to bind to ultrafine particles in the bloodstream and are most likely associated with physiological clearance processes [49]. Most available literature showed either no change in IgG levels or a slight increase. These studies were, however, done in sensitized experimental animal models, therefore more research on human participants will be necessary in order to provide a basis for a clinical significance of this finding [50, 51].

A clinical significance of these findings is arguable. In terms of haemostasis, Gilmour et al. [52] previously examined but could not find effects of ultrafine and fine carbon black particle exposure using similar UFP concentrations as measured in our study. Recent data indicates an association between exposure to UFPs (and larger particles) and both, acute-phase proteins and pro-coagulation parameters, in the same time span considered in our study, which was described as a possible link between air pollution and cardiovascular events [53–56]. Negative correlations between environmental particulate air pollution (PM_10_) and haemoglobin concentration, packed cell volume, red blood count (*p* < 0.001), platelets and factor VII levels (*p* < 0.05), were reported [57] previously, but did not match our results. Data on systemic effects of UFP exposure is in fact controversial in literature, probably depending on the study design, studied population, particle type, model used and questions asked.

The content of lead in blood was significantly higher in the exposed subjects than in the control group (109.33 ± 103.63 (SD) μg/L vs. 36.24 ± 20.42 μg/L; *p* = 0.0008), which not only shows that metal particles were indeed incorporated but also that the incorporation exceeded the recommended doses (in Austria maximum blood lead concentrations should be below 90 μg/L in male workers), putting them at higher risk for adverse health effects associated with higher blood lead levels [58].

In occupational settings the concentration and size of UFPs depends not only on the (technological) process, but also on the efficacy of the exhaust system. Efficient exhaust systems predominantly reduce large particle load of the breathing air. Unexpectedly, at two other typical occupational settings with high burdens of UFPs, welding and laser beam application on a glass coating, Riediger and Möhlman [59] have demonstrated that by switching the local exhaust system on, the number of UFPs is reduced for 1 to 1,5 orders of magnitude. In our study, we did not measure the number and size of particles when the exhaust system was turned off because of ethical considerations.

### Study limitations

There are some limitations to our study. Firstly, we initially measured submicrometer sized particles (< 700 nm), but since we did not find adverse respiratory effects and monodispersed particles ranged between 58.85 ± 16.94 and 98.19 ± 22.83 nm in our measurements, we did not calculate the exact concentrations of UFP (< 100 nm). Secondly, a possible limitation for the lack of adverse effects found in our study could be the absence of reactive metals, since it has been shown before, that oxidative stress induced by UFPs depends on the content of reactive metals. Thirdly, gender differences could not be assessed in this study due to the lack of exposed female subjects, but men may be more resistant to UFPs related adverse health effects than women. Further there were only three time points of air flow measurements: before, 4 h and 24 h after exposure. Thus, short-term changes following exposure to UFPs might have been missed. Finally, the young age of our subjects, as well as their overall good health and relatively short continuous exposure to UFPs at the workplace (4.7 years on average) might have been limiting study factors.

## Conclusions

We could not detect consistent indications for adverse respiratory or inflammatory effects directly following exposure and 24 h after exposure to high levels of submicrometer and UFPs in our study group. These findings imply that high levels of submicrometer sized airborne particle concentrations, with a predominant fraction of UFPs, do not induce significant airway inflammation in healthy male subjects. The value of this study is the assessment of the short-term exposure effects to UFPs at a genuine occupational setting, which might is relevant when a risk assessment of high level occupational exposures to UFPs is considered. The implication, furthermore is, that our study supports the theory of a threshold value under which no observed effects level (NOEL) could be found by conventional diagnostic measures in otherwise healthy workers. Long-term prospective studies evaluating the effects of occupational UFPs exposure on human health are however required for the development of reasonable safety measures and preventive work-place related medical surveillance methods.
